# GRUSP, an Universal Stress Protein, Is Involved in Gibberellin-dependent Induction of Flowering in *Arabidopsis thaliana*

**DOI:** 10.1134/S1607672921040062

**Published:** 2021-08-23

**Authors:** D. S. Gorshkova, I. A. Getman, L. I. Sergeeva, Vl. V. Kuznetsov, E. S. Pojidaeva

**Affiliations:** 1 Timiryazev Institute of Plant Physiology, Russian Academy of Sciences, Moscow, Russia; 2 Moscow State University, Faculty of Biology, Moscow, Russia; 3 Wageningen University & Research, Wageningen, The Netherlands

**Keywords:** flowering, phytohormones, universal stress protein, *At3g58450*, GRUSP

## Abstract

The effect of T-DNA insertion in the 3'-UTR region of *Arabidopsis thaliana At3g58450* gene encoding the Germination-Related Universal Stress Protein (GRUSP) was studied. It was found that under a long-day condition this mutation delays transition to flowering of *grusp-115* transgenic line that due to a reduced content of endogenous bioactive gibberellins GA1 and GA3 in comparison to the wild-type plants (Col-0). Exogenous GA accelerated flowering of both lines but did not change the time of difference in the onset of flowering between Col-0 and *grusp-115*. In addition to changes in GA metabolism, *grusp-115* evidently has disturbances in realization of the signal that induces flowering. This is confirmed by the results of gene expression of the floral integrator *FLOWERING LOCUS T* (*FT*) and the floral repressor *FLOWERING LOCUS C* (*FLC*), which are key flowering regulators and acting opposite. We hypothesize that the formation of *grusp-115* phenotype can also be affected by a low expression level of *FT* due to up-regulated *FLC* expression.

The transition to the reproductive stage is the most important stage of the flowering plant ontogenesis. For the long-day (LD) plant *Arabidopsis thaliana* (L.) Heynh, gibberellins (GA) are the main phytohormones controlling induction of flowering [1], and these ones are also strictly necessary under short-day (SD) conditions [2]. Under LD, gibberellins are indirectly involved in flowering induction through the regulation of expression and functioning of the main floral integrators of the photoperiodic flowering pathway FLOWERING LOCUS T (FT) and SOC1. In leaves, accumulation of GA leads to degradation of transcription repressors of the DELLA group and induces transcription of *FT* gene [3, 4] by the activation of *CO* and *SPL* transcription factors [5]. In the apical meristem, the GA-dependent degradation of DELLA proteins leads to activation of LEAFY and SOC1 expression, which, in turn, provides a transition to flowering [6].

Besides gibberellins, abscisic acid (ABA) plays a significant role in flowering. Depending on growth conditions and the plant physiological state, the effect of ABA can both accelerate and delay the transition from vegetative to a reproductive stage of ontogenesis [7]. In conditions of long day, ABA-dependent transcription factors ABI4 and ABI5 underlie the late flowering [8, 9]. ABI4 inhibits transition to flowering by the repression of GA biosynthesis and stimulating the expression of floral repressor FLOWERING LOCUS C (FLC) [8–10]. Additionally, the DELLA proteins promote activation of FLC, thereby providing a close relationship with GA-dependent responses [11].

GRUSP protein (Germination-Related Universal Stress Protein) encoded by *At3g58450* gene *A. thaliana* [12, 13] is a potential member in pathways controlling the balance between ABA and GA throughout different growth stages. *At3g58450* expression significantly increases in Col-0 seedlings treated with ABA, and impairment of its transcription causes ABA hypersensitivity of transgenic seeds *grusp-115* [12].

The aim of this study is to analyze for the first time the relationship of a late-flowering phenotype of *grusp-115* transgenic line, characterized by suppressed expression of *At3g58450* due to T-DNA insertion in the 3'-UTR, with the endogenous gibberellins content, as well as with the expression of genes that control the transition to reproductive development.

In this study, we used *A. thaliana* (L.) Heynh ecotype Columbia wild type (Col-0) and *GABI_kat 115C08* (*grusp-115*) homozygous transgenic line [12]. Plants were grown in a chamber with a controlled environment at 16-h photoperiod, light intensity of 100 μmol m^–2^ s^–1^ and temperature +21°C.

The development of Col-0 and *grusp-115* plants was analyzed according to the scale of growth stages, which is used to identify and interpret phenotypic differences and to determine specific growth stages in *A. thaliana* as previously described [14]. The beginning of the generative stage was indicated by the appearance of an inflorescence head in a rosette. Flowering time was measured as the number of days after stratification or the number of rosette leaves at the time of flower buds emergence.

The content of bioactive GA was detected in 10 mg lyophilized samples obtained from the rosette leaves of 4-week-old Col-0 and *grusp-115* plant [15]. From studies using an isotope label, it is known that exogenously applied GAs are effectively absorbed by leaves and subsequently transported to the shoot apex in their bioactive form [16]. In this connection, the response of plants to exogenous GAs was studied using a 100 μM aqueous solution of GA_4 +7_ (Sigma, USA). For this, 14-day-old seedlings were sprayed with 100 μM GA supplemented with 0.02% (v/v) Tween-20 twice a week for 12 weeks. Control plants were sprayed with a water solution containing only 0.02% Tween-20.

The transcript levels of *FLC* (*At5g10140*) and *FT* (*At1g65480*) genes were estimated by the real-time PCR (qRT-PCR) as described previously [13]. For amplification of gene fragments, pairs of primers were used: *FLC* 5'-AAAGTAGCCGACAAGTCACC-3' and 5'-GGATGCGTCACAGAGAACAG-3'; *FT* 5'-GCCAGAACTTCAACACTCGC-3' and 5'-AGCCACTCTCCCTCTGACAA-3'. All experiments were carried out in 3-fold biological replication. Significance in differences was tested using the Student’s test (*t*-test).

It was found that the delay in germination of *grusp-115* seeds is the result of changes in expression level of GA metabolism genes, including gibberellins biosynthetic genes, *GA20ox1* and *GA3ox1* [13]. Decreased expression of genes involved in GA biosynthesis can also be the reason for the longer vegetative growth stage observed for *grusp-115* (Fig. 1a).

**Fig. 1.**
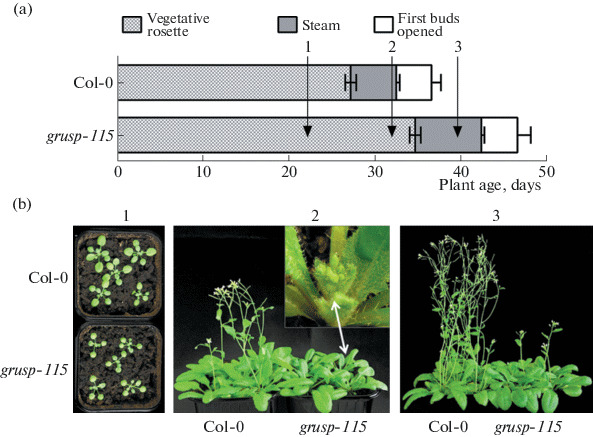
Characterization of Col-0 and *grusp-115* plants growth under long-day conditions: (a) diagram of plant development according to Boyes [14]. The numbers indicate the age of plants whose rosette leaves were collected for further qRT-PCR analysis of *FT* and *FLC* transcript levels: (1) 23-, (2) 34-, and (3) 42- days of growth after stratification; (b) phenotype of (1) 23-, (2) 34- and (3) 42-day-old plants.

In Col-0, a clearly visible inflorescence head was formed 28–30 days after stratification, while in *grusp-115*, a delay in inflorescence formation was observed up to 35 days. Moreover, the stem elongation in Col-0 began 3–5 days after the emergence of a flower bud, while in *grusp-115,* this did not occur, and the head of inflorescence remained longer inside of rosette leaves (Fig. 1b; number 2 corresponds to 35-day-old plants). Full-sized flowering shoots with siliques were formed in 45- day-old Col-0 plants, while *grusp-115* at this age was at the stage of shoot elongation and opening of the first flower buds (Fig. 1b, number 3 is 45- day-old plants). Thus, the time difference between Col-0 and *grusp-115* was 10 days since the inflorescence formation and shoot elongation. Subsequently, *grusp-115* formed a semi-dwarf shoot with siliques changed in length and shape, with a small number of seeds (Fig. 1b). Full-fledged shoots with developed pods were formed only after 50–55 days.

Under LD, at least two factors, photoperiodism and gibberellins, may play a key role in the transition from vegetative to generative stage of development [4]. In this case, the photoperiodic pathway plays a pivotal role in induction of flowering, but GAs perform an auxiliary function [4].

Our results demonstrate that a later flowering of *grusp-115*, probably due to the reduced content of bioactive GA1 and GA3 (Fig. 2) and can be restored by applying exogenous GA (Fig. 3). The number of rosette leaves is an important parameter of *A. thaliana* growth and development [2, 14]. In conditions of LD, Col-0 forms 10–14 rosette leaves and then the shoot apical meristem is rearranged into the inflorescence meristem [14]. In our study, during vegetative growth, Col-0 plants formed on average 11 rosette leaves, while *grusp-115* started flowering in only about 14 leaves (Fig. 3a). The treatment of plants with exogenous GAs accelerated the transition to reproductive stage and was observed for Col-0 plants that possess not 11, but only 9 leaves. The *grusp-115* showed a similar reaction to GA treatment as a wild type (Fig. 3b) and started flowering after 9 rosette leaves were formed (Fig. 3a). Thus, the delay in transition to flowering, expressed in a number of rosette leaves, was completely eliminated in *grusp-115* after treatment of plants with exogenous gibberellins. GA-dependent recovery of a late-flowering phenotype of *grusp-115* mutant to the wild type phenotype confirms the idea that the transgenic line is deficient in endogenous GA (Fig. 2). At the same time, GA treatment did not eliminate time differences (by 10 days) in transition to flowering of *grusp-115* compared to Col-0 (Fig. 3a).

**Fig. 2.**
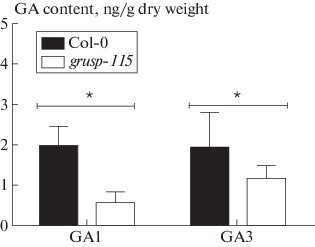
The content of bioactive gibberellin GA1 and GA3 in 4-week-old vegetative rosette tissues of Col-0 and *grusp-115* plants. An asterisk denote statistically significant differences between Col-0 and *grusp-115* according to Student’s *t*-test (* *p* < 0.1).

**Fig. 3.**
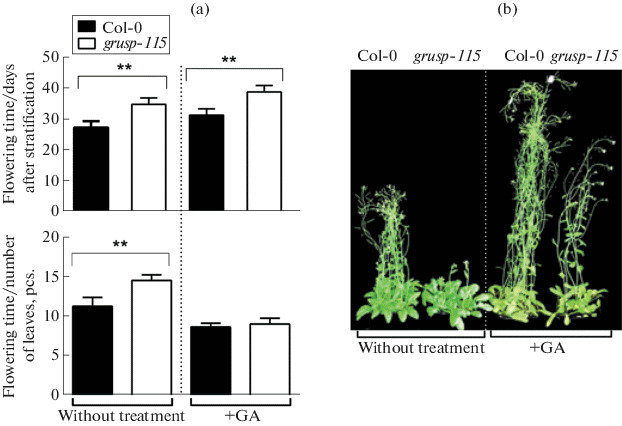
Response of Col-0 and *grusp-115* plant to application of 100 μM exogenous gibberellins GA_4+7_: (а) flowering time expressed in the number of days after stratification and the number of rosette leaves at the time of bud appearance in the rosette; (b) phenotype of plants before and after GA treatment via 42 days of growth. An asterisks indicate statistically significant differences between Col-0 and *grusp-115* according to Student’s *t*-test (** *p* < 0.05).

To search for other possible reasons for a temporary backlog in the transition to flowering between Col-0 and *grusp-115*, the expression levels of key floral genes *FT* and *FLC* were analyzed in rosette leaves of both lines at different development stages (Fig. 4). For this purpose, rosette leaves of plants of 23, 34, and 42- day-old indicated as numbers 1, 2, and 3, respectively, were used. The *FT* gene was expressed at a low level in leaves of any age (Fig. 4), but with maximum intensity at the age of 42 days (3), which corresponds to a flowering plant with formed inflorescences (Fig. 1b). Mutant *grusp-115* showed a reduced expression level of *FT* gene both at the stage of vegetative growth at the age of 23 days (1) and at the stage of transition to flowering at the age of 42 days (Fig. 4; (3)).

**Fig. 4.**
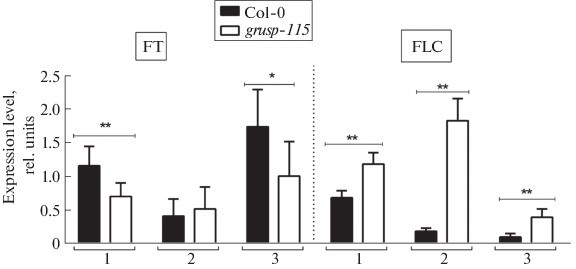
Relative expression level of *FT* and *FLC* genes in Col-0 and *grusp-115* rosette leaves. Samples were taken at different stages of plant growth, depending on their age: 23 (1), 34 (2), and 42 (3) days after stratification. An asterisks denote statistically significant differences between Col-0 and *grusp-115* according to Student’s *t*-test (* *p* < 0.1, ** *p* < 0.05).

The flowering repressor FLC, which suppresses the expression of a number of floral genes, including *FT*, inhibits the transition to reproductive development stage [6]. Figure 4 shows that *FLC* expression level is higher in *grusp-115* at all studied growth stages (1–3; Fig. 1a) than in Col-0. The maximum difference was observed at 34 days (2), when the Col-0 plants already had flowering shoots and the *grusp-115* plants were still in a vegetative state. Moreover, the level of *FLC* transcripts was elevated in *grusp-115* even after the appearance of flower buds (3), which explains here the decreased expression of *FT* gene.

In this work we demonstrated for the first time that the basis for a later transition to flowering of *grusp-115* T-DNA insertion line compared to Col-0 of *A. thaliana* is a reduced content of bioactive endogenous GA1 and GA3, as well as a lower expression of *FT* gene due to increased accumulation of the flowering repressor *FLC*. We assume that GRUSP protein is a new regulatory component of flowering signal transduction pathways.
